# Evaluation of changes arising in the pig mesenchymal stromal cells transcriptome following cryopreservation and Trichostatin A treatment

**DOI:** 10.1371/journal.pone.0192147

**Published:** 2018-02-01

**Authors:** Artur Gurgul, Joanna Romanek, Klaudia Pawlina-Tyszko, Tomasz Szmatoła, Jolanta Opiela

**Affiliations:** 1 National Research Institute of Animal Production, Department of Genomics and Molecular Biology, Balice, Poland; 2 National Research Institute of Animal Production, Department of Animal Reproduction Biotechnology, Balice, Poland; EFS, FRANCE

## Abstract

Cryopreservation is an important procedure in maintenance and clinical applications of mesenchymal stem/stromal cells (MSCs). Although the methods of cell freezing using various cryoprotectants are well developed and allow preserving structurally intact living cells, the freezing process can be considered as a severe cellular stress associated with ice formation, osmotic damage, cryoprotectants migration/cytotoxicity or rapid cell shrinkage. The cellular response to freezing stress is aimed at the restoring of homeostasis and repair of cell damage and is crucial for cell viability. In this study we evaluated the changes arising in the pig mesenchymal stromal cell transcriptome following cryopreservation and showed the vast alterations in cell transcriptional activity (5,575 genes with altered expression) suggesting the engagement in post-thawing cell recovery of processes connected with cell membrane tension regulation, membrane damage repair, cell shape maintenance, mitochondria-connected energy homeostasis and apoptosis mediation. We also evaluated the effect of known gene expression stimulator—Trichostain A (TSA) on the frozen/thawed cells transcriptome and showed that TSA is able to counteract to a certain extent transcriptome alterations, however, its specificity and advantages for cell recovery after cryopreservation require further studies.

## Introduction

Mesenchymal stem cells (MSCs) are highly attractive for tissue engineering and clinical applications because of their inherent regenerative capacity, high proliferation potential, immunomodulatory activity, ability to differentiate into different cell lineages and low immunogenicity [1]. In most methodologies, MSCs are enriched from bone marrow aspirates by density gradient centrifugation [2], but their amount usually is insufficient for further procedures. Isolation protocols resulting in high initial cell counts are available and desirable for one-step procedures in regenerative medicine [3, 4], however, they are cost and time-consuming. This makes *in vitro* expansion of undifferentiated MSCs an indispensable procedure for both scientific and application purposes. *In vitro* expansion, however, carries the risk of contamination by pathogens or malignant transformation and in addition, the multilineage differentiation capacity of stem cells may be lost during long-term expansion [5]. In various application procedures, cryopreservation plays an important role in obtaining off-the-shelf availability for cells. It also separates cell culture from application and prepares cells for long distance transportation and long term storage.

Cryopreserved cells are likely to be the main cell source for tissue engineering and stem cell therapy [6, 7]. Thus, development of technologies which allow storing and banking of MSCs with minimal loss of cell viability, differentiation capacity and function has been under active investigation. It was found that cryopreservation can affect differentiation ability of stem cells [8, 9] and cause the loss of a variety of pluripotency markers [10, 11], but exact reasons for these changes remain elusive. On the other hand, several studies using MSCs derived from different tissues and cryopreserved with 10% Me_2_SO applying slow freezing protocols showed that the frozen MSCs maintained similar phenotypes, cell surface markers and growth rates in comparison to freshly cultured cells [12, 13]. A fast freezing protocol employing vitrification have also been investigated, showing normal proliferation, phenotype and differentiation of MSCs [14, 15]. Nevertheless, cryopreservation and cell storage can be considered as environmental stress [16], which can be mediated through one or a combination of different factors, such as cytotoxicity of cryoprotective agents [17], osmotic injury caused by the excursion of a cryoprotective agent upon a freeze-thawing cycle [18], intracellular ice formation during the cooling process [19] and re-crystallization of the intracellular ice during the warming process [20]. Long term storage of cryopreserved human PBMCs resulted in disturbances of transcript levels of 1,367 genes, whose expression after 14 months was affected >3 fold following isolation, cryopreservation and thawing as compared to freshly isolated PBMC [21]. The cryopreservation-induced stress was also described in fibroblasts grown in three-dimensional culture, where it induced a specific cellular stress response involving growth factors [16]. Moreover, it is also assumed that cryopreservation may disturb epigenetic mechanisms associated with cell development and differentiation. In studies investigating cryopreserved zebrafish genital ridges or human spermatozoa a methylation level of some genes was found to be altered after a freezing-thawing procedure. Also the transcript levels of important pluripotency factors like *pou5f1* and *sox2* were found to be altered after cryopreservation [22].

Maintenance of stemness of adult stem cells is to a large extent governed by unique combinations of epigenetic regulators [23]. Epigenetic mechanisms, including histone acetylation/deacetylation, play a crucial role in transcriptional regulation via remodeling of chromatin architecture [24]. Histone deacetylases (HDACs), classified into four classes [25, 26] catalyze a wide spectrum of physiological processes including proliferation, differentiation, apoptosis and cell cycle regulation [27]. One of commonly used culture media factors which was shown to interfere with histones acetylation and stabilize the expression of pluripotent genes is Trichostain A (TSA). TSA is an organic compound that serves as an antifungal antibiotic and selectively inhibits the class I and II mammalian HDACs [28]. It was found that human MSCs treated with a low dosage of TSA *ex vivo* show increased expression of pluripotent genes such as *Oct4*, *Sox2*, *Nanaog*, *Rex-1* and *TERT*. Administration of low concentrations of TSA also significantly suppressed morphological changes in MSCs occurring during culture expansion, increased their proliferation potential and at the same time retained their contact inhibition properties and multipotent differentiation abilities [29].

According to the above, in this study we hypothesized that TSA, through its epigenetic activity, may efficiently counteract the effect of cryopreservation stress in pig bone-marrow derived MSCs. To verify this hypothesis, we attempted to evaluate changes in MSC gene expression profiles caused by a freezing-thawing procedure and evaluated the result of TSA addition on profile of genes expression in these cells. We additionally performed a quantitative analysis of genes associated with critical biological processes affected either by cryopreservation or TSA administration and evaluated their transcriptional activity in cells harvested at different culture stages including: control fresh MSC, MSC subjected to cryopreservation, TSA-treated MSC and MSC cultured another 24 h after TSA removal.

## Methods

### Animals

Five outbred Polish Large White (PLW) pigs of both sexes, weighing approximately 20 kg each, were maintained under conventional conditions in the pigsty in the Department of Animal Reproduction Biotechnology at the National Research Institute of Animal Production in Balice, Poland. The veterinary care was provided by the institution. All animal procedures were approved by the Local Animal Care Ethics Committee No. II in Kraków–permission number 1256/2015 in accordance with EU regulations.

### Isolation and in vitro culture of pig mesenchymal stromal cells (pMSCs)

The pig mesenchymal stromal cells (MSCs) were isolated as described earlier [30]. In brief, the bone marrow was aspirated from the iliac crests of the animals under general anaesthesia (Thiopental i.v.- 0.01g/kg). The animals after the procedure were provided with analgesics (Pyralgin i.m. -15-50 mg/kg) and anti-inflammatory and antibacterial agents (Betamox i.m.– 0.1 ml/kg). Mononuclear cells were separated by gradient centrifugation at 400 x g for 20 min over a layer of Ficoll-Paque Plus (Stem Cell Technologies; SCT, Canada) and cell suspensions were seeded at a concentration of 1x 10^5^ cells/cm^2^ per 75 cm^2^ culture flask (Corning, USA) containing 17 ml medium comprised of low-glucose Dulbecco's modified Eagle's medium (DMEM; Sigma-Aldrich, Germany) supplemented with 10% foetal bovine serum (FBS; Sigma-Aldrich, Germany) and 1% antibiotic-antimycotic solution (AAS; Sigma-Aldrich, Germany). The cells were incubated at 39°C and 5% CO_2_ in 100% humidified air. Non-adherent cells were removed after the first 24 h. The culture medium was changed every 3 days thereafter. When cells reached about 80–90% confluence, they were detached and digested into single-cell suspensions using 0.25% trypsin-0.01% EDTA solution (Sigma-Aldrich, Germany) and either cryopreserved in 10% Me_2_SO/FCS (a detailed description below) or suspended in 100 μl PBS and snap-frozen in liquid nitrogen (LN2) to lyse cells. The snap-frozen MSCs in PBS constituted the non-cryopreserved control group (CTR).

### The expression of surface antigens typical for MSCs by flow cytometry and MSC differentiation

The detailed analysis of surface antigens (positive and negative) typical for MSCs by flow cytometry was described in our latest paper [30].

The differentiation of MSCs into osteoblasts and adipocytes was described by Opiela et al. [31].

### Cryopreservation and thawing of pMSCs

After dissociation with 0.25% trypsin-EDTA solution, MSCs were washed twice with HEPES supplemented with 10% FBS by centrifugation at 500×g for 5 min, and then 0.5 x number of passaged flasks milliliters of 10% Me_2_SO/FCS was added to the washed cells as the freezing medium. The cells suspended in 0.5 mL of the freezing medium were transferred into 1.0 mL cryovials (Thermo Scientific, Roskilde, Denmark) which were directly transferred to the freezer (-20°C) and stored for 2 hours. Then, the cryovials were quickly dipped into LN_2_ and stored for 2 weeks before thawing.

The cryopreserved MSCs of five pigs were thawed at 37°C in a water bath with gentle shaking and the cryoprotective solution was removed by gradual dilution with TCM199-HEPES medium supplemented with 10% FBS. The cells were centrifuged at 500×g for 5 min to remove the freezing medium. Then, the cells from each pig were re-suspended in three flasks filled with 17 mL of pMSCs medium (low-glucose DMEM) enriched in 10% foetal bovine serum (FBS), 1% antibiotics and/or 1% Glutamax (Invitrogen, USA) at the density of approx. 1x 10^4^ cells/cm^2^ for *in vitro* culture. The cells were allowed to attach and TSA was added to the medium of two flasks and incubated for 24 h when the confluency reached 60–70%. After that time, the medium was discarded, the cultures were terminated by trypsinisation as described above and the cells were suspended in 100 μl PBS without Ca and Mg followed by snap freezing in LN2 to lyse the cells. The remaining third flask of each pig was further cultured for additional 24 h. By such procedure we obtained three experimental groups, namely: 1. The cryo control group—frozen/thawed MSCs *in vitro* cultured in DMEM supplemented with 5%FCS (Cryo); 2. The TSA group—frozen/thawed MSCs *in vitro* cultured in DMEM supplemented with 5%FCS and 50nM TSA for 24 h (TSA); 3. The TSA>24 group—frozen/thawed MSCs *in vitro* cultured in DMEM supplemented with 5%FCS and 50nM TSA for 24 h followed by 24 h of *in vitro* culture in DMEM/5%FCS without TSA (TSA>24). The cultures were terminated by trypsinisation as described above and the cells were suspended in 100 μl PBS without Ca and Mg followed by snap freezing in LN2 to lyse the cells. The cryovials were stored in LN2 for 2 to 4 weeks before a RNA-seq analysis.

### Analysis of frozen/thawed MSC specific markers (CD73, CD90, CD105) by Western-blotting

MSCs of three different pigs, cryopreserved in 10% Me_2_SO/FCS when the round shape colonies were already formed and stored few weeks in liquid nitrogen were thawed and re-plated into T75 culture flasks. The cells were *in vitro* cultured in DMEM supplemented with 5% FCS till reaching 70% confluence. Then, each biological sample was divided into three treatments: 1. *in vitro* culture in DMEM supplemented with 5% FCS (Cryo); 2. *in vitro* culture in DMEM supplemented with 5% FCS and 50nM TSA (TSA); 3. *in vitro* culture in DMEM supplemented with 5% FCS and 50nM TSA for 24 h followed by 24 h in DMEM/5% FCS (TSA>24h). On the day of the experiment, samples were solubilized in tissue protein extraction reagent T-PER (Thermo Fisher Scientific) containing a protease inhibitor cocktail (539134, Merck, Warsaw, Poland). Samples were sonicated for 10 sec in an ultrasonic cell disrupter (TORBEO, 36810; Cole—Parmer, Vernon Hills, USA) after 5 min incubation. The denatured samples were separated by electrophoresis on a discontinuous SDS gel system consisting of 4% polyacrylamide stacking and 12% separating components. Then, proteins were transferred from the gels onto 0.22 mm PVDF membranes in 10 mM CAPS containing 10% methanol using a semidry electroblotting apparatus (Bio-Rad, Warszawa, Poland) at 50 V for 50 min. The membranes were blocked with TBS-T (25 mM Tris, 0.5 M NaCl, pH 7.5 + 0.1% Tween 20) blocking buffer and supplemented with 0.2% (w/v) skim milk powder. Blocking, washing and incubations with primary and secondary antibodies were performed at room temperature in the Snap id system (Merck). The blocked membranes were incubated for 10 min at room temperature with primary antibodies for CD73, CD90 and CD105 (Santa Cruz Biotechnology, Inc, USA). The description of antibodies and the used concentrations are provided in Table 1.

**Table 1:** The antibodies used for Western-blotting and their concentrations.

Protein/antigen	Antibody	concentrations	
		primary antibody- goat polyclonal	secondary antibody- anti-goat HRP-labelled antibody
CD73	sc-14682	63 μg/ mL	0.1 μg/mL
CD90	sc-6071	21 μg/ mL	0.1 μg/mL
CD105	sc-19793	21 μg/ mL	0.1 μg/mL
actin	sc-1615	0.2 μg/mL	0.1 μg/mL

The membranes were washed four times in TBS-T and incubated for 10 min at room temperature with the appropriate secondary antibody (Santa Cruz Biotechnology, Inc, USA) conjugated to horseradish peroxidase (HRP) at a specific concentration. The membranes were washed again three times with TBS-T. Subsequently, the proteins were visualized using a chemiluminescence kit (Pierce Biotechnology, Inc. Rockford, IL, USA) on Fusion Solo.6S.WL according to the manufacturer’s instructions (Vilber Lourmat, Marne La Vallée, France). The images were scanned, and the optical density (OD) of the bands was measured using Fusion Solo.6S.WL. For re-probing with actin as the loading control, the membrane was washed in stripping buffer (100 mM 2-mercaptoethanol, 20% SDS, 62.5 mM Tris—HCl, pH 6.7) at 56°C for 60 min to strip off the bound antibody. After washing in TBS-T and blocking, the membrane was re-probed with the goat polyclonal anti-actin antibody (sc–1615, Santa Cruz Biotechnology, Inc, USA) (0.2 μg/mL) for 10 min at room temperature. The secondary anti-goat HRP-labelled antibody (sc–2020, Santa Cruz Biotechnology, Inc, USA) (0.1 μg/mL) was incubated for 10 min at room temperature. Actin was visualized as previously described by chemiluminescent reaction. The images were scanned, and the optical density (OD) of the bands was measured using Fusion Solo.6S.WL (Vilber Lourmat, Marne La Vallée, France). The ratio of the OD of analyzed proteins relative to actin is presented in the form of bar charts. The significant differences in protein levels were assessed by Tukey's post–hoc one–way ANOVA.

### Library preparation and transcriptome sequencing

Total RNA was purified from harvested cells using Direct-zol^™^ RNA MiniPrep Kit (Zymo Research, CA, USA) and controlled in terms of quality using Agilent TapeStation2200 system. Only samples with RNA Integrity Number (RIN) above 8 were used for further analysis. In total, 500 ng of RNA was used for library construction with the TruSeq RNA Sample Prep v2 kit (Illumina, San Diego, CA). Standard library preparation steps including: mRNA selection, fragmentation, cDNA synthesis, end repair, adenylation, indexed adapters ligation and amplification were followed by a qualitative evaluation (Agilent TapeStation 2200) and quantitation (Qubit, Thermo Fisher Scientific). The validated, normalized and pooled libraries were ultimately sequenced in a single 50-bp indexed run (1 x 50 bp) on the HiScanSQ system using TruSeq SBSv3 Sequencing kit (Illumina). Raw reads were deposited in NCBI Sequence Read Archive (SRA) database under BioProject number PRJNA395970.

### Data analysis

The obtained raw reads were controlled for quality using FastQC software and filtered/trimmed with Flexbar software [32]. The filtering allowed removing reads with phred quality score below 20 and read length below 30nt after adapter trimming. The filtered reads were mapped against the *Sus scrofa10*.*2* genome with the TopHat2 software [33] set to single-end mode and Bowtie “sensitive” option. The reads mapped to exons were counted using HTSeq software in ‘Union’ mode [34], providing Ensembl GTF annotation file v10.2.87. Data normalization and a differential expression analysis were performed using DESeq2 software [35] in pairwise comparisons among the study groups. The quality of the RNA-Seq read mapping was controlled using RseQC software [36]. Differences in gene expression profiles among the groups were evaluated using principal component analysis (PCA) and hierarchical clustering. The adjusted p-value <0.05 (after FDR correction using the Benjamini-Hochberg procedure, q-value) was used as a cutoff for differentially expressed (DE) genes. To allow direct comparison of gene-specific read counts when analyzing separate genes (pluripotency, differentiation markers and others), raw reads count tables for individual samples were normalized together in an additional step for all the study groups using DESeq2.

The detected DE genes were evaluated in terms of their biological functions and modulated pathways with respect to gene ontology (GO) terms in PANTHER Classification System [37]. Additional GO and KEGG pathway analyses were performed using KOBAS 3.0 web server [38] or WebGestalt toolkit (WEB-based GEne SeT AnaLysis Toolkit [39]). Only processes with similar biological significance found to be overrepresented by at least two of these algorithms were considered. All the overrepresentation/enrichment tests were performed with respect to all known genes and with a correction for multiple testing.

### qPCR validation

Validation of the obtained results was carried out with the use of RT-qPCR method. First, primers specific for seven chosen genes including five pluripotency marker genes with low, medium and high expression levels (*KLF4*, *FGF2*, *LIF*, *TERT*, *NANOG*), one neurogenic differentiation marker (*NSE*) and one highly expressed SOX family member (*SOX9*) were designed (S1 File). Then, High-Capacity cDNA Reverse Transcription kit (Thermo Fisher Scientific) was used to reverse transcribe selected RNA samples (with sufficient amount of RNA left after the RNA-Seq analysis). AmpliQ 5x HOT EvaGreen^®^ qPCR Mix Plus (ROX) (Novazym) was used to run qPCR reactions according to the manufacturer instructions. The reactions were run in triplicates for each sample including melting curve analysis on QuantStudio^™^ 7 Flex Real-Time PCR System (Thermo Fisher Scientific). To determine endogenous control that is a gene with the most stable expression, obtained data were analyzed using NormFinder software [40]. Next, ΔΔCt method corrected for reaction efficiency (E) calculated on the basis of standard curves was applied to quantify relative expression levels [41]. The RNA-Seq results comparison with qPCR was based on evaluation of correlation coefficients between expression level of each gene across samples determined with both methods and across genes (averaged expression per gene).

## Results

### The expression of surface markers typical for MSCs estimated by flow-cytometry in MSCs not subjected to freezing

We proved by flow cytometry the mesenchymal origin of the porcine bone-marrow derived cells. The results are already published by Gurgul et al. [30]. The analysis was performed on isolated MSCs and TSA-modulated and unmodulated MSCs not subjected to freezing/thawing procedure. Moreover, we proved the mesenchymal origin of the porcine bone-marrow derived cells by performing their differentiation into osteoblasts and adipocytes [31].

### The expression of positive surface markers typical for MSCs estimated by Western-blotting in frozen/thawed MSCs

**Fig 1:**
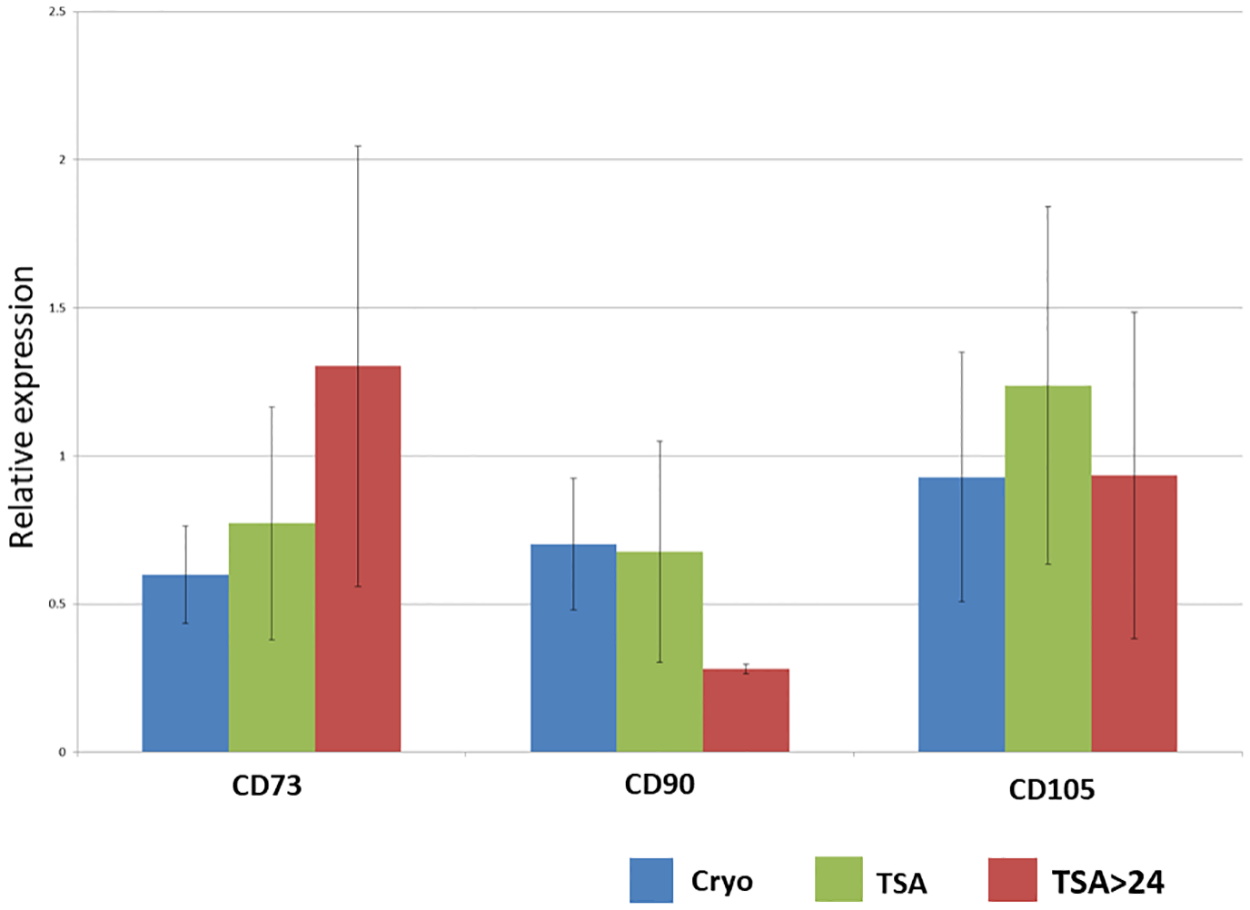
Densitometry of CD73, CD90 and CD105 proteins expression in MSCs from three different treatments: 1- cryo; 2- TSA; 3- TSA>24. Cryo–cryopreserved, not treated cells TSA–cells treated with TSA for 24h TSA>24h –cells treated with TSA for 24h and further cultured following TSA removal.

**Fig 2:**
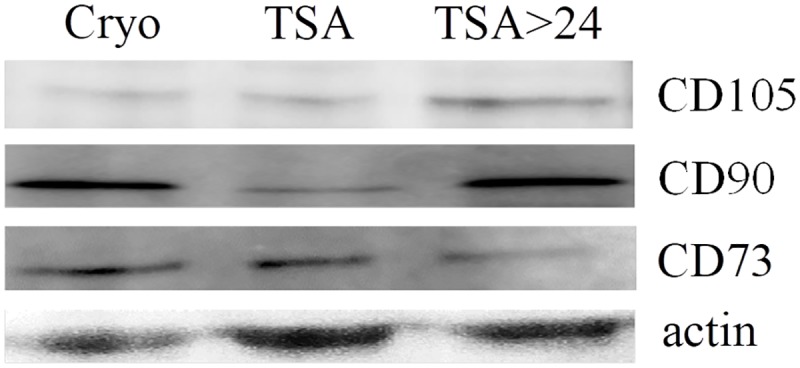
Exemplary protein bands after the Western blot analysis of CD105, CD90, CD73 and actin in MSCs from three different treatments: Cryo, TSA and TSA>24. Cell lysates were subjected to SDS-PAGE and analyzed by Western blotting. The membrane was probed with an antibody against CD73. The same blot was reprobed with an antibody against actin. The same procedure was repeated for estimating CD90 and CD105. Cryo–cryopreserved, not treated cells TSA–cells treated with TSA for 24h TSA>24h –cells treated with TSA for 24h and further cultured following TSA removal.

Frozen control MSCs (cryo group) and MSCs subjected to TSA following cryopreservation (TSA and TSA>24h) were evaluated for the expression of CD73, CD90 and CD105 proteins by SDS-PAGE and immunoblotting with polyclonal antibodies anti-CD73, anti-CD90 and anti-CD105 (Figs 1 and 2).

The cells of all the analyzed groups expressed the typical MSC markers confirming their multipotent character. Moreover, no significant differences were noted in CD73, CD90 and CD105 proteins expression in MSCs from three treatment groups implying that TSA has neither positive nor negative impact on these markers' expression (Figs 1 and 2).

### Sequencing reads statistics

**Fig 3:**
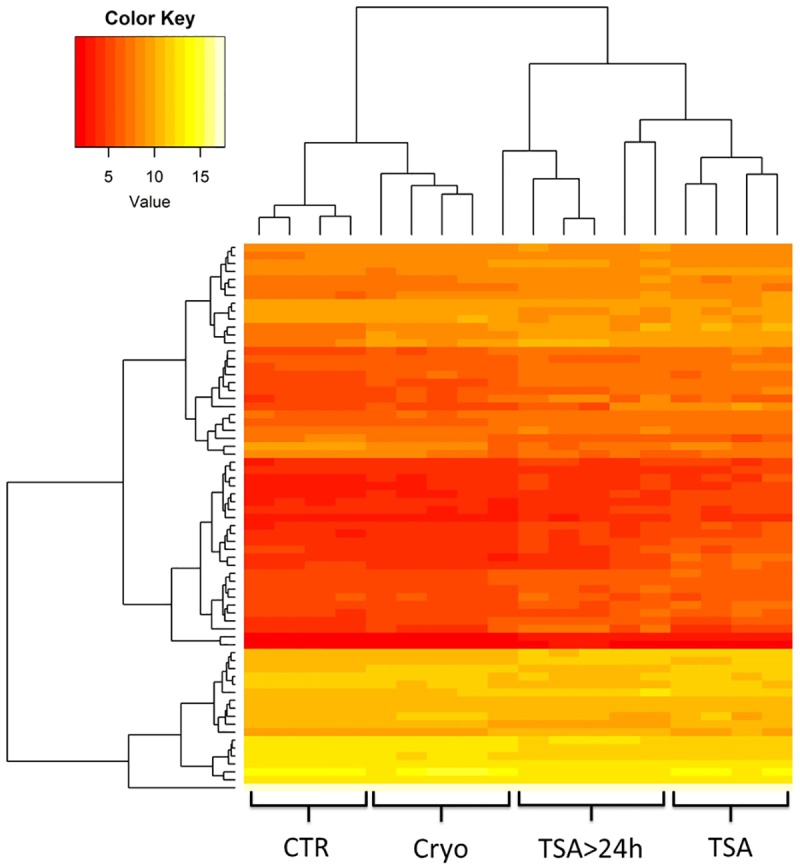
Hierarchical clustering of expression profiles of all studied cultures based on genes with the highest differentiation among the groups. CTR–fresh (not cryopreserved) control Cryo–cryopreserved, not treated cells TSA–cells treated with TSA for 24h TSA>24h –cells treated with TSA for 24h and further cultured following TSA removal.

The RNA-Seq libraries belonging to four study groups encompassing four to five MSC cultures obtained from five different pigs were sequenced in single-end 50-bp run, which resulted in about 564.4 million of raw reads. The number of reads generated per sample ranged from 24.0 to 46.6 M with a mean for separate groups ranging from 27.6 to 34.1 M. On average, 84% of filtered reads were successfully mapped against the reference genome and 13.3% of these reads had multiple mappings. The comparable number of expressed genes (with mean normalized read counts >1) was detected for the control, frozen-thawed and cultured after TSA removal MSCs (about 13.4 thousand), with a slightly higher number of expressed genes observed in the frozen TSA-treated cells (14.2 thousand) (Table 2). The analysis of expression profiles of all analyzed cultures using hierarchical clustering with genes showing the highest variation among the study groups revealed a general expression profile similarity within the groups and visible inter-group differentiation. Expression profiles of two cultures prolonged after TSA removal (TSA>24h) showed similar, but still distinct, profile of expression in comparison to the cells harvested after 24h of TSA treatment (Fig 3).

**Table 2:** Sequencing reads and mapping statistics for all analyzed samples.

Sample	Group	Number of raw reads	Number of reads after filtering	% of mapped reads	% of reads with multiple mappings	Number of reads uniquely mapped to genes	% of reads uniquely mapped to genes in regard to all reads mapped	Number of detected genes (normalized read count >1)
47	Control	33,527,496	33,232,716	87.0	14.6	19,281,625	66.7	13,402
52		29,337,818	29,051,179	85.8	14.5	16,554,887	66.4	14,074
54		32,501,711	32,245,946	86.5	14.6	18,459,014	66.2	14,246
55		36,884,314	36,708,672	86.9	14.7	21,185,195	66.4	13,547
All (mean)		33,062,835	32,809,628	86.6	14.6	18,870,180	66.4	13,817
9	Cryo	39,811,022	39,383,308	86.5	12.3	21,009,157	61.7	13,537
21		31,911,754	31,657,966	86.4	12.4	17,096,115	62.5	13,776
23		26,988,514	26,751,854	83.8	14.3	14,613,012	65.2	13,894
26		28,433,528	28,101,216	84.9	13.2	15,698,595	65.8	13,950
37		24,011,188	23,860,067	73.8	13.3	11,877,827	67.5	13,682
All (mean)		30,231,201	29,950,882	83.1	13.1	16,058,941	64.5	13,768
19	TSA	32,611,524	32,221,390	86.9	11.5	17,572,194	62.8	14,406
27		26,944,001	26,583,205	83.1	12.3	14,832,174	67.1	14,539
28		26,311,428	25,950,421	81.2	12.4	14,101,883	66.9	14,575
32		24,388,215	24,162,907	80.6	12.6	13,234,780	68.0	13,329
All (mean)		27,563,792	27,229,481	83.0	12.2	14,935,258	66.2	14,212
4	TSA>24h	34,013,910	33,511,242	85.8	13.5	17,274,048	60.0	13,536
5		37,985,222	37,506,718	86.0	13.6	19,971,007	61.9	13,653
10		46,651,711	46,251,409	86.0	13.3	25,326,901	63.7	14,036
25		26,118,944	25,933,779	80.5	12.9	14,034,619	63.2	14,101
38		25,941,128	25,863,891	81.0	12.5	14,025,411	66.9	14,150
All (mean)		34,142,183	33,813,408	83.9	13.2	18,126,397	63.1	13,895
All (mean)		31,354,079	31,054,327	84.0	13.3	17,008,247	64.9	13,913

### Effect of cryopreservation on the pig MSCs transcriptome

**Fig 4:**
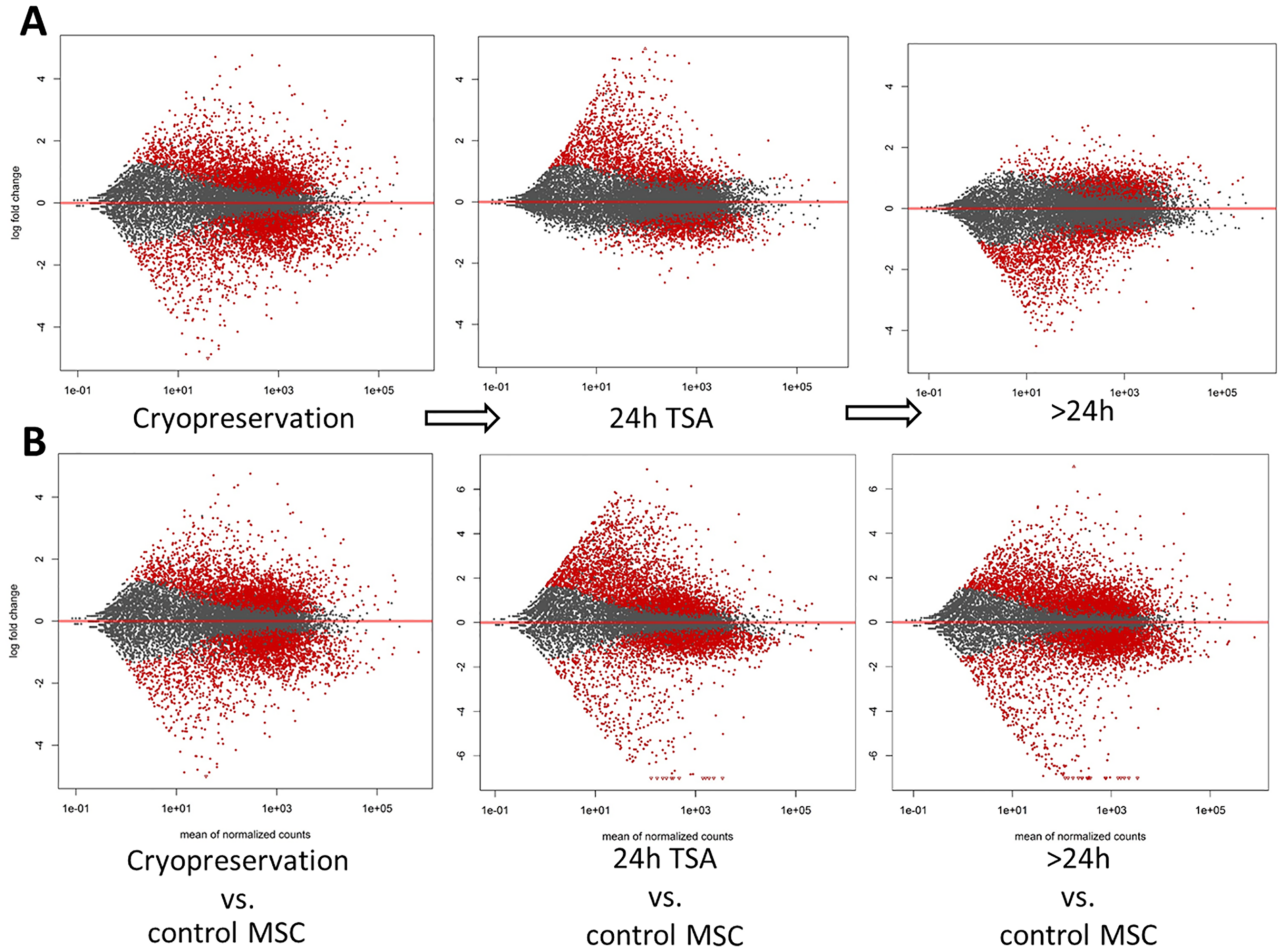
Log_2_ fold change and the mean expression level for all genes in the studied MSCs. Significantly differentially expressed genes are marked in red, A- in comparison to preceding treatment; The genes expression level was compared between the frozen and non-frozen control, the TSA treated cells and frozen control and the cells further cultured following TSA removal vs. the TSA treated cells; B- in comparison to the fresh (non-frozen) MSCs.

The effect of a freezing-thawing procedure on the pig MSC transcriptome was evaluated based on cells obtained from five cryopreserved and four control cultures. The comparison of general expression profiles of the frozen and control cells across the genome (measured as a correlation coefficient between mean expression level of all genes in each group), suggested that the cryopreservation introduced significant changes in behavior of several genes (r = 0.837; p<0.01). Further differential gene expression analysis showed extensive alterations in the MSC transcriptome manifesting by significant up- or downregulation of 5,575 different genes (with genome-wide q<0.05). Almost equal number of the genes was up- and downregulated in the frozen cells with the average absolute log_2_fold change of 1.09 (±0.642) (Fig 4).

To minimize the number of DE genes and allow a more detailed analysis of their biological functions, the top 1000 DE genes with the lowest adjusted p-values were selected (p<1.0E-05) (S2 File). Among these genes, 391 were upregulated with the mean log_2_FC of 1.54 and the remaining ones were downregulated with a similar order of magnitude of log_2_FC (-1.61). The analysis of physiological implications of the genes aiming at the identification of major affected cellular processes, showed that the upregulated genes significantly enriched molecular functions associated with e.g. cytoskeletal protein binding or actin binding and were mainly related to biological processes involved in cell adhesion, biological adhesion, cellular component organization and regulation of cell communication. The upregulated genes also enriched several biological processes responsible for negative regulation of general biological or cellular processes and positive regulation of intracellular protein transport, protein import into nucleus and nucleocytoplasmic transport. Cellular components enriched with the upregulated genes encompassed mainly integral components of membrane and those localized in cell periphery, but also adherens and anchoring junctions, focal adhesions or stress fibers (Table 3). The analysis of Panther, KEGG and Reactome pathways showed that the upregulated genes enriched pathways that are involved in integrin signaling (P00034), Wnt signaling (P00057) tight junction (ssc04530), regulation of actin cytoskeleton (ssc04810), inflammatory mediated regulation of TRP channels (ssc04750) and pathways in cancer (ssc05200).

**Table 3:** Summary of biological processes, molecular functions and cellular components affected by genes upregulated in the post-thawing MSC culture.

GO	Upregulated		
	term ID	Description	log10 p-value
BP	GO:0016043	cellular component organization	-3.9172
	GO:0051606	detection of stimulus	-3.7212
	GO:0007606	sensory perception of chemical stimulus	-3.3696
	GO:0071840	cellular component organization or biogenesis	-3.0975
	GO:0007043	cell-cell junction assembly	-2.5901
	GO:0048519	negative regulation of biological process	-2.0277
	GO:0048523	negative regulation of cellular process	-2.0277
	GO:0032879	regulation of localization	-2.0277
	GO:0090136	epithelial cell-cell adhesion	-2.0277
	GO:0048518	positive regulation of biological process	-2.0214
	GO:0045444	fat cell differentiation	-1.7194
	GO:0051049	regulation of transport	-1.71
	GO:0034330	cell junction organization	-1.6216
	GO:0022610	biological adhesion	-1.5352
	GO:0010646	regulation of cell communication	-1.5287
	GO:0023051	regulation of signaling	-1.3768
MF	GO:0005515	protein binding	-10.0132
	GO:0005488	binding	-5.2118
	GO:0019899	enzyme binding	-4.0635
	GO:0005509	calcium ion binding	-3.2118
CC	GO:0031982	vesicle	-2.6108
	GO:0071944	cell periphery	-2.3412
	GO:0012505	endomembrane system	-2.1791
	GO:0043226	organelle	-2.0804
	GO:0030054	cell junction	-1.8601
	GO:0043227	membrane-bounded organelle	-1.7595
	GO:0005925	focal adhesion	-1.7061
	GO:0005886	plasma membrane	-1.7061
	GO:0005575	cellular component	-1.6882
	GO:0044444	cytoplasmic part	-1.6778
	GO:0001725	stress fiber	-1.5009
	GO:0005856	cytoskeleton	-1.4787

Numerous enriched biological processes were analyzed with Revigo system to summarize them by removing redundant GO terms. Not all detected GO categories are displayed.

Molecular functions enriched with the genes downregulated after cryopreservation included mainly those associated with translation (e.g. structural constituent of ribosomes, RNA/poly(A) RNA binding) or energy homeostasis (e.g.: electron carrier activity, NADH dehydrogenase activity, cytochrome-c oxidase activity, ATP binding and general oxidoreductase activity). The genes enriched molecular function-related biological processes, including inter alia: translation, gene expression, ribosome biogenesis, nitrogen compound metabolic processes, hydrogen ion transmembrane transport, ATP metabolic process, oxidative phosphorylation, respiratory electron transport chain or purine ribonucleotide metabolic processes. The top enriched cellular components in which the downregulated genes manifest their functions, involved mainly: mitochondrion, mitochondrial envelope, mitochondrial inner membrane, ribosome, intracellular ribonucleoprotein complex, and cytoplasmic cell part. The genes downregulated after cryopreservation enriched several pathways of which the most interesting seem to be: ribosome (ssc03010), oxidative phosphorylation (ssc00190), respiratory electron transport, ATP synthesis by chemiosmotic coupling, and heat production by uncoupling proteins (R-SSC-163200), nonsense-mediated decay (NMD) (R-SSC-927802), mitochondrial translation (R-SSC-5368287) or translation initiation complex formation (R-SSC-72649) pathways (Table 4).

**Table 4:** Summarized biological processes, molecular functions and cellular components affected by genes downregulated in the post-thawing MSC culture.

GO	Downregulated		
	term ID	description	log10 p-value
BP	GO:0006807	nitrogen compound metabolic process	-9.8539
	GO:0010467	gene expression	-9.2684
	GO:1901564	organonitrogen compound metabolic process	-8.8477
	GO:0006518	peptide metabolic process	-8.2034
	GO:0044271	cellular nitrogen compound biosynthetic process	-7.9547
	GO:0043603	cellular amide metabolic process	-7.8697
	GO:0006412	translation	-7.8297
	GO:0007600	sensory perception	-7.5867
	GO:1901566	organonitrogen compound biosynthetic process	-7.1337
	GO:0051606	detection of stimulus	-7.0013
	GO:0007186	G-protein coupled receptor signaling pathway	-6.8665
	GO:1902600	hydrogen ion transmembrane transport	-6.7905
	GO:0009593	detection of chemical stimulus	-6.1824
	GO:0007154	cell communication	-5.8097
	GO:0009058	biosynthetic process	-5.7825
	GO:0042254	ribosome biogenesis	-5.6126
	GO:0032501	multicellular organismal process	-5.5591
	GO:0023052	signaling	-5.3768
	GO:0044237	cellular metabolic process	-4.8539
	GO:0022900	electron transport chain	-4.6778
	GO:0008152	metabolic process	-3.8539
	GO:0016072	rRNA metabolic process	-3.6576
	GO:0009145	purine nucleoside triphosphate biosynthetic process	-3.3969
	GO:0055086	nucleobase-containing small molecule metabolic process	-3.0269
	GO:0006888	ER to Golgi vesicle-mediated transport	-2.7011
	GO:0065007	biological regulation	-2.5969
	GO:0071704	organic substance metabolic process	-2.475
	GO:0006123	mitochondrial electron transport, cytochrome c to oxygen	-2.3582
	GO:0046483	heterocycle metabolic process	-2.0159
	GO:0006091	generation of precursor metabolites and energy	-1.8725
	GO:0002181	cytoplasmic translation	-1.7457
	GO:0050896	response to stimulus	-1.7235
	GO:0019637	organophosphate metabolic process	-1.6281
	GO:0006725	cellular aromatic compound metabolic process	-1.5482
	GO:0044238	primary metabolic process	-1.5072
MF	GO:0003735	structural constituent of ribosome	-300
	GO:0004871	signal transducer activity	-8.0899
	GO:0060089	molecular transducer activity	-8.0283
	GO:0005198	structural molecule activity	-7.6737
	GO:0015078	hydrogen ion transmembrane transporter activity	-7.6737
	GO:0009055	electron carrier activity	-4.857
	GO:0016675	oxidoreductase activity, acting on a heme group of donors	-4.0862
	GO:0003723	RNA binding	-4.0862
	GO:0015002	heme-copper terminal oxidase activity	-4.0862
	GO:0003954	NADH dehydrogenase activity	-2.9788
	GO:0043565	sequence-specific DNA binding	-2.6108
	GO:0044822	poly(A) RNA binding	-2.4621
	GO:0016679	oxidoreductase activity, acting on diphenols and related substances as donors	-2.4621
	GO:0016651	oxidoreductase activity, acting on NAD(P)H	-2.3958
	GO:0016491	oxidoreductase activity	-2.33
	GO:0001071	nucleic acid binding transcription factor activity	-1.8861
	GO:0003700	sequence-specific DNA binding transcription factor activity	-1.8861
	GO:0005524	ATP binding	-1.6596
CC	GO:0032991	macromolecular complex	-300
	GO:0098800	inner mitochondrial membrane protein complex	-300
	GO:0022626	cytosolic ribosome	-300
	GO:0044444	cytoplasmic part	-36.5638
	GO:0005737	cytoplasm	-36.2299
	GO:0043226	organelle	-33.466
	GO:0005622	intracellular	-30.1965
	GO:0044464	cell part	-13.4685
	GO:0005623	cell	-13.1965
	GO:0031975	envelope	-11.8894
	GO:0098796	membrane protein complex	-11.341
	GO:0070469	respiratory chain	-9.0862
	GO:0005829	cytosol	-7.9101
	GO:0005575	cellular_component	-5.9914
	GO:0070069	cytochrome complex	-4.6946
	GO:0071944	cell periphery	-4.4134
	GO:0005886	plasma membrane	-4.118
	GO:1903561	extracellular vesicle	-4.066
	GO:0097526	spliceosomal tri-snRNP complex	-3.4949
	GO:0000313	organellar ribosome	-2.9252
	GO:0034709	methylosome	-2.7481
	GO:0031982	vesicle	-2.5719
	GO:0034719	SMN-Sm protein complex	-2.5119
	GO:0005732	small nucleolar ribonucleoprotein complex	-2.5119
	GO:0019773	proteasome core complex, alpha-subunit complex	-1.8316
	GO:0031974	membrane-enclosed lumen	-1.8186
	GO:0005793	endoplasmic reticulum-Golgi intermediate compartment	-1.4973
	GO:0000502	proteasome complex	-1.4973
	GO:0032040	small-subunit processome	-1.3255

Numerous enriched biological processes were analyzed with Revigo system to summarize them by removing redundant GO terms. Not all detected GO categories are displayed.

### Effect of TSA administration on the transcriptome of cryopreserved MSCs

The culture of cryopreserved MSCs with 50mM TSA for 24 h resulted in significant changes in transcript levels of lower number of genes than in the case of cryopreservation itself, nevertheless, as many as 2759 genes were affected, of which about 58% were upregulated. The absolute average log_2_ fold change for the upregulated genes was about two times higher than for the downregulated (1.61 vs. 0.81) (Fig 4). The correlation coefficient between the mean gene expression levels in cryopreserved cells and TSA treated cells across the genome was 0.981 (p<0.01), suggesting extensive changes in gene expression profiles. To analyze in details biological significance of the DE genes and to find the major affected processes, we again focused on the top 1000 genes with the lowest adjusted p-value (<1.0E-04) (S3 File). Of the genes, 699 were upregulated with the average log_2_FC of 2.21. The downregulated genes (n = 301) were characterized by a lower fold of change, which was reflected in the value of log_2_FC equal to -1.01. Biological functions enriched with TSA-stimulated genes were roughly similar to those previously observed on fresh MSCs (Gurgul et al., 2017) and involved inter alia: neuron projection development, single-multicellular organism process, nervous system development, neuron development and general developmental process. These biological processes were convergent with molecular functions and cellular components enriched with the genes which included e.g. ion channel activity, neuron part, synapse part, synapse or integral components of membrane.

The genes downregulated by TSA after 24h culture of cryopreserved cells enriched only very few GO categories, however, they could be connected with the processes like: endoplasmic reticulum functioning, intracellular protein transport, vesicle coat synthesis or G-protein coupled receptor activity.

**Fig 5:**
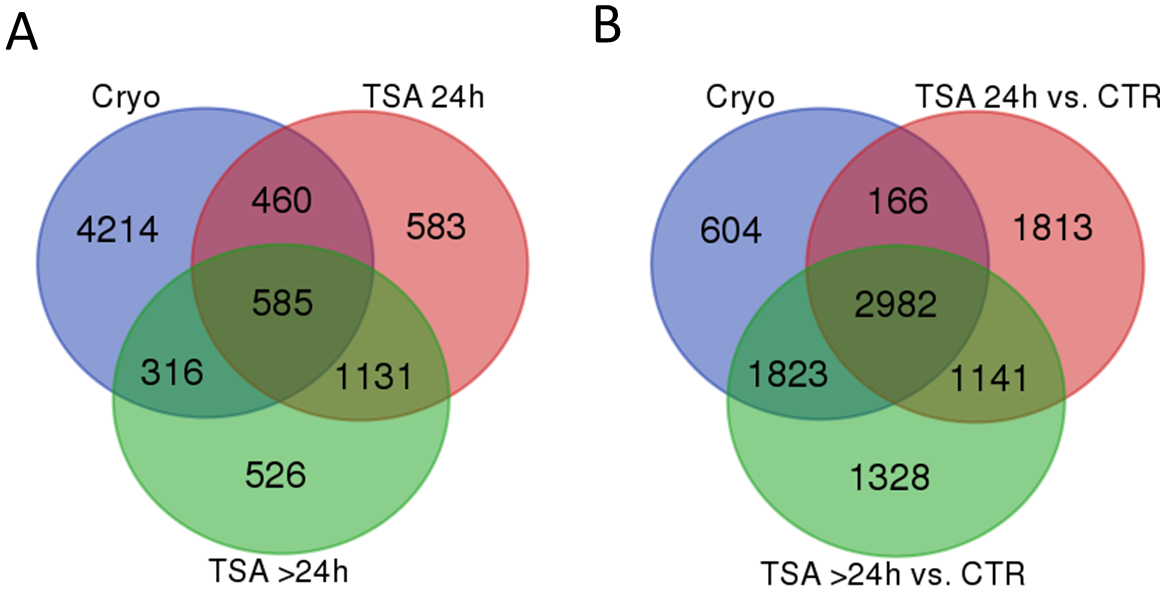
Venn diagram of differentially expressed genes between–A- preceding treatments and B- fresh control MSCs. A—The genes expression level was compared between the frozen and non-frozen control, the TSA treated cells and frozen control and the cells further cultured following TSA removal vs. the TSA treated cells; B- in comparison to the fresh (non-frozen) MSCs. CTR–fresh (not cryopreserved) control Cryo–cryopreserved, not treated cells TSA–cells treated with TSA for 24h TSA>24h –cells treated with TSA for 24h and further cultured following TSA removal.

A comparison of genes differentially expressed after MSCs cryopreservation with those further altered by TSA submission showed 1045 common entries (Fig 5). They included 539 genes initially upregulated by cryopreservation stress of which 330 were reversely regulated by TSA. These genes mainly enriched biological processes associated with hydrogen ion transmembrane transport, oxidation-reduction or single-organism metabolic process. The remaining genes initially upregulated by cryopreservation and further stimulated by TSA treatment enriched only a scarce number of categories of biological processes connected with: cells morphogenesis involved in neuron differentiation and cellular components morphogenesis. Transcripts which were suppressed by cryopreservation and their suppression was deepened by TSA administration belonged to 98 genes with functions enriched in biological processes connected with inter alia: intracellular protein transport, establishment of localization or Golgi vesicle transport. Another set of 408 genes which were suppressed by cryopreservation and further upregulated by TSA addition enriched biological processes associated with e.g. cytokinesis, intracellular protein transport or response to stimulus.

A separate analysis was performed to detect genes whose expression was affected by cryopreservation and then restored to the control level after TSA treatment (i.e. differentially expressed after cryopreservation but not after TSA treatment of cryopreserved cells in comparison to the fresh control cells). The gene list consisted of 2427 entries of which comparable numbers were up- (n = 1286) and downregulated (n = 1141) with similar log_2_ fold of change (0.86 vs. -0.93). The genes which were initially downregulated by cryopreservation and upregulated to the control level by TSA administration or natural cell mechanisms, showed overrepresentation in a wide range of biological processes of which the highest number of detected genes was connected with: metabolism (e.g. regulation of cellular metabolic process; regulation of macromolecule metabolic process; regulation of cellular biosynthetic process), gene expression (e.g. regulation of nucleic acid-templated transcription; regulation of RNA biosynthetic process; regulation of transcription from RNA polymerase II promoter), development (e.g. system development; anatomical structure morphogenesis; nervous system development) or cellular components organization (organelle organization; cellular component organization). The genes were overrepresented in different classes of cellular components associated mainly with: nucleus (nucleus; nucleoplasm; nuclear lumen), intracellular organelles (intracellular organelle; intracellular organelle lumen; intracellular membrane-bounded organelle) or cell membrane (membrane part; plasma membrane; membrane-enclosed lumen). The mostly enriched Reactome pathways included: generic transcription pathway (R-SSC-212436), gene expression (R-SSC-74160) and signal transduction (R-SSC-162582) pathways.

Genes that were initially upregulated by cryopreservation and returned to the control level (downregulated) after 24h TSA treatment enriched biological processes in categories associated with: metabolism (e.g. cellular metabolic process; organic substance metabolic process; primary metabolic process; peptide metabolic process); signal transduction and signaling (signal transduction; single organism signaling; cell communication), energy homeostasis (ATP metabolic process; oxidation-reduction process; respiratory electron transport chain; oxidative phosphorylation) or purine nucleoside/ribonucleotide metabolism and synthesis (e.g. purine ribonucleoside biosynthetic process; purine nucleoside biosynthetic process; purine ribonucleotide metabolic process). Molecular functions enriched with the highest number of genes were connected mainly with oxidoreductase activity, poly(A) RNA binding and catalytic activity, but also with NADH dehydrogenase activity, ATPase activity or RNA polymerase activity. Cellular components in which the downregulated genes were overrepresented included a broad range of cellular compounds from membrane to cytoplasm and organelles with several categories associated with mitochondria.

### Changes in the expression profile of genes involved in major biological processes associated with cryopreservation and TSA treatment

**Fig 6:**
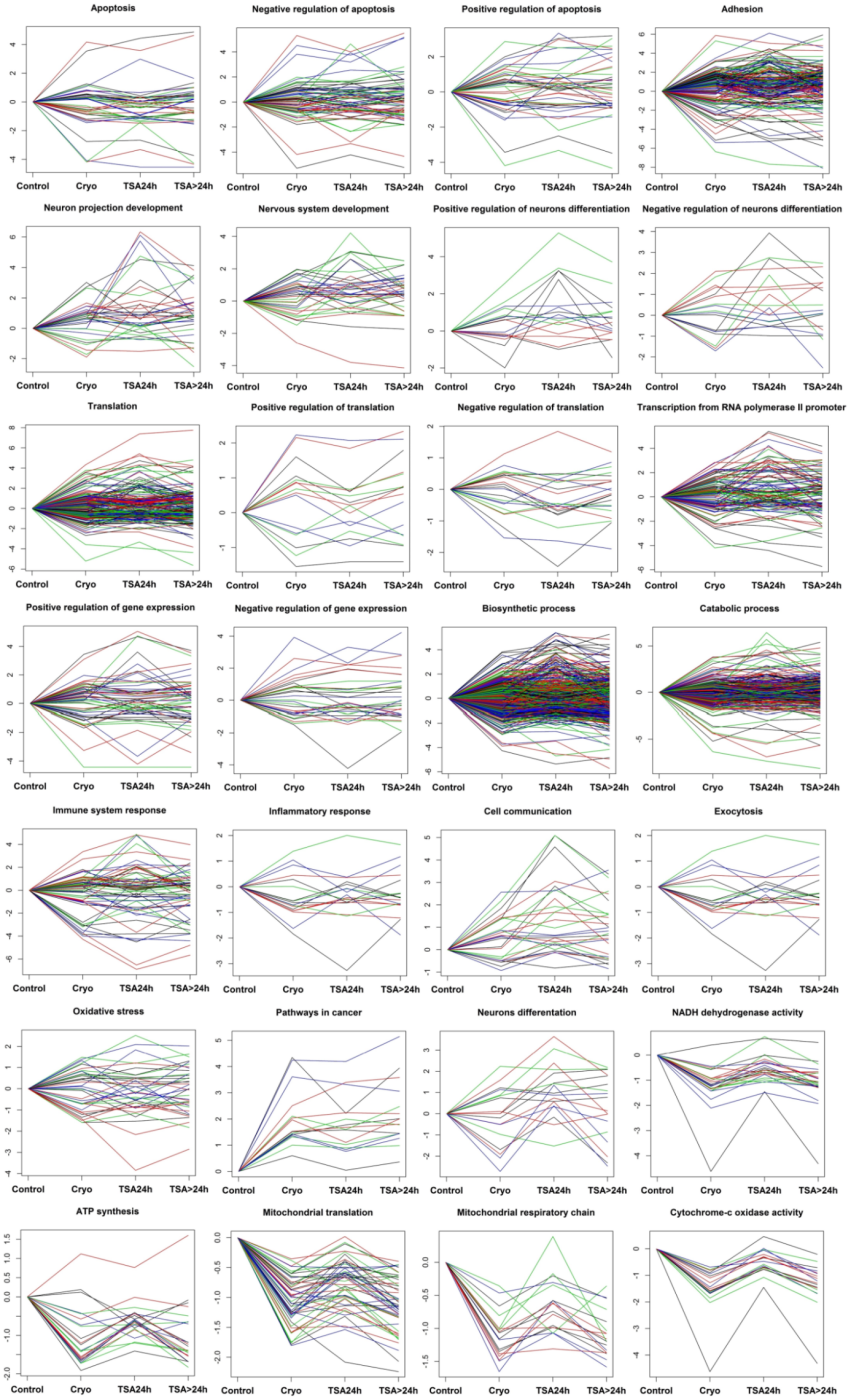
Changes in expression level of selected differentially expressed genes with division into major processes affected by cryopreservation and TSA treatment. The normalized read counts for all samples were averaged, log_2_ transformed and presented as deviations from the expression level observed in fresh control MSCs.

The identification of major biological processes and pathways affected by cryopreservation and TSA administration allowed us to look closer into changes in expression profiles of differentially expressed genes classified with respect to specific altered processes. Expression levels of the genes were evaluated at all four culture conditions (time points), and involved: control fresh MSCs, MSCs subjected to cryopreservation, TSA-treated MSCs and MSCs cultured another 24h after TSA removal. Based on results from the whole-genome analysis, as major classes of biological processes corresponding to cryopreservation stress we selected those which were found to be significantly enriched and had the highest number of involved genes, namely: adhesion, translation, oxidative stress, ATP synthesis, mitochondrial translation, gene expression and cell communication. The major processes affected by TSA treatment found in this study encompassed neurons differentiation and neuron projection development. Additionally, we considered genes engaged in known cellular responses to cryopreservation stress or malignant transformation, such as: apoptosis, inflammatory response and pathways in cancer. Genes engaged in previously described processes [30] affected by TSA administration, like e.g.: exocytosis and immune system response were also included (Fig 6). In most of these processes, cryopreservation and subsequent TSA administration induced noticeable changes in gene expression profiles involving both up- and downregulation without easy to evaluate pattern. Nevertheless, interesting results were obtained when expression profiles of genes engaged in processes responsible for: energy homeostasis (ATP synthesis, mitochondrial respiratory chain, cytochrome-c oxidase activity and NADH dehydrogenase activity) and mitochondrial translation were analyzed in details. The engaged genes were mostly strongly downregulated by cryopreservation, but subsequent TSA treatment exerted an opposite stimulating effect on most of these genes which ceased after TSA removal. Cryopreservation also visibly stimulated transcription of majority of genes engaged in cell communication or pathways in cancer. The succeeding TSA administration also showed some signs of suppression of genes involved in carcinogenesis but further stimulated genes engaged in cell communication. In accordance with our previous findings, the coordinated effect of TSA addition on gene expression manifested mainly through stimulation of genes associated with neurogenesis (neuron projection development, nervous system development, positive/negative regulation of neurons differentiation) and cell communication. This effect, however, ceased after TSA removal (Fig 6).

### Effect of cryopreservation and further TSA treatment on pluripotency/multipotency markers expression in pig MSCs

**Fig 7:**
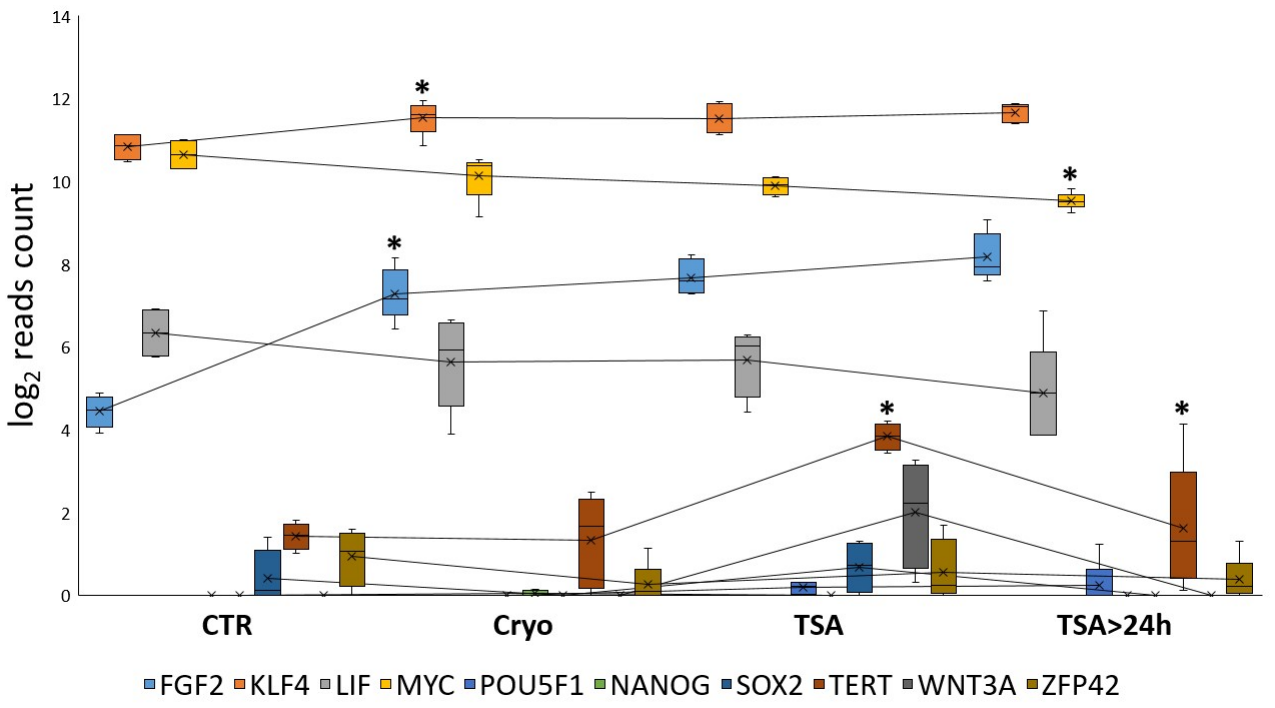
Changes in expression level of major pluripotency marker genes after cryopreservation, TSA administration and TSA removal. Significant differences in expression level on pointwise level are marked with asterisk.

To evaluate the effect of applied experimental conditions on the expression level of known pluripotency markers such as *FGF2*, *KLF4*, *LIF*, *c-MYC*, *POU5F1*, *NANOG*, *SOX2*, *TERT*, *WNT3A* and *ZFP42*, normalized counts for each of the genes were compared between the separate treatments. Across all the study groups, with the achieved sequencing depth, virtually no expression was found for *POU5F1*, *NANOG*, *SOX2*, *WNT3A* genes while a low expression level was detected for *TERT* and *ZFP42*. The remaining pluripotency-associated genes had intermediate to high transcript levels with some minor changes introduced by a specific treatment. *FGF2* and *KLF4* expression was significantly elevated (p<0.05 at a pointwise level) after cryopreservation and remained increased after TSA administration or TSA removal. *c-MYC* was characterized by a gradually decreasing expression level with a significant transcript loss after prolonged culture without TSA (p<0.05). A distinct profile of expression was observed for *TERT* gene, whose expression was elevated after TSA treatment and downregulated in further culture after TSA removal (Fig 7).

### Validation of the results using RT-qPCR

The obtained RNA-seq results were validated using an alternative method–RT-qPCR. Among the chosen genes, *NANOG* gene was characterized by the most stable expression therefore it served as an endogenous control. The correlation coefficient between expression level of individual validated genes across samples was satisfying for four out of six analyzed genes and ranged from 0.30 for *KLF* gene to 0.94 for *FGF2* (with a mean of 0.72). For two other genes (*NSE*, *TERT*) correlation coefficient was low or negative suggesting some results bias. Nevertheless, the comparative analysis of expression levels of genes (averaged expression) estimated using both methods showed a satisfying concordance between the RNA-Seq and qPCR results, which correlated with r = 0.69, suggesting no significant bias in relative expression estimation for the analyzed genes (S1 File).

## Discussion

Cryopreservation is currently a standard procedure in most applications involving *in vitro* cell expansion and their preservation for a further use. Cryopreservation allows the storage of cells for prolonged time periods while maintaining them in adequate condition for use in clinical or research settings. Freezing and storing of cells are also standard procedures in reproductive biotechnology where gametes or embryos are cryopreserved and stored for long time while maintaining their viability and developmental abilities. Conventional cryopreservation systems include slow-freezing and vitrification, both having advantages and disadvantages in terms of cell viability and/or scalability [42]. In this study we used a simplified freezing procedure. Cryovials with cells suspended in a cryoprotectant were stored for 2 h in -20°C and then snap-frozen in liquid nitrogen. We observed that after re-placing in culture the cells maintained their phenotype although part of them did not attach which is a normal observation as around 1/5 of the frozen cells dies during the freezing/thawing procedure [43]. This method of freezing has been routinely performed in our laboratory to freeze somatic cells [44, 45] and mesenchymal stem cells [46] used as donors of nuclei for somatic cell cloning purposes as well as VERO cells used as co-culture cells for bovine and goat embryo *in vitro* culture [47, 48]. Presently the cooling protocol of MSCs from various tissues and species is mostly based on controlled freezing [49] although different approaches of cryopreservation of porcine MSC are rather scarcely described compared to other species. Our experiment, due to extensive results on porcine transcriptome, delivers convincing evidence for the simple 2-step freezing procedure to be effective and efficient. There are reports on successful freezing results in other species where a MSCs cooling protocol was not based on controlled freezing using a freezer: e.g. human MSC [50, 51, 52, 53], monkey MSC [54] and dog MSC [55].

Although the effect of cryopreservation on *in vitro* cultured cell phenotypes was widely studied, research on changes arising in the global cell transcriptome is scarce, especially for non-human cell models. Knowledge on gene expression changes emerging as a result of cryopreservation-induced cell damage or environmental stress may be useful for identification of major cellular processes affected by freezing-thawing procedures and development of more efficient cryopreservation methods or culture additives counteracting cell viability loss. The initial results of this study suggested that cryopreservation induces rather a decrease in global transcriptional activity of cells, thus we assumed that culture additives such as TSA (acting rather as a gene expression stimulator) may counteract transcriptional changes induced by cryopreservation and enable faster cell recovery after cryopreservation stress. In the final results the relative transcript loss after freezing and further culturing turned out not to be as large as expected, however, involved downregulation of more than two thousand genes. So, apart from evaluating the effect of cryopreservation on the pig bone-marrow derived MSC transcriptome we also decided to test the influence of TSA on transcriptional activity of frozen cells.

The results of this study showed that the process of MSC cryopreservation with the applied method induced changes in the expression level of more than five thousand genes in further culture of which almost equal numbers were down- or upregulated. The number of affected genes is difficult to compare with results of other research because comprehensive genome-wide studies analyzing changes in the gene expression profile after cryopreservation are scarce, especially for *in vitro* cultured cell lines. One of the available studies showed that the expression of 1,367 genes was altered after 14 months of frozen cells storage with >3 fold change in human PBMCs. Moreover, more than three thousand genes were found to be affected with fold of change >2 in these cells [21]. Another available study also showed a large number of altered genes in frozen-thawed human embryonic stem cells and demonstrated that the number of the upregulated genes reaches a maximal plateau within 24h of culture after thawing, and drops down at 48h. The number of the downregulated genes increases gradually along with the time of culturing [56]. The almost equal number of up- and downregulated genes found in this study conform to the above mentioned results because our sampling of cells was carried out after 24h of post-thawing culture.

To analyze in details cellular processes associated with genes affected by cryopreservation, the number of differentially expressed genes was reduced to an arbitral amount of one thousand genes with the highest statistical significance of differences in the expression level. Although using a fold change as an additional cutoff in DE analysis is a commonly used method for narrowing down gene lists, we decided to rely only on p-value, considering that a differentially expressed at a statistically significant level gene needs to be clearly quantitatively different between the biological groups and relatively consistent within each group of replicates. This allowed us to retain genes with high expression profile uniformity within the groups which may be more characteristic for the applied conditions than changes in the expression level of genes with a high fold change and clear within-group variability [57].

Among the selected genes, 39.1% were upregulated by cryopreservation. The analysis of those genes in terms of their functions, allowed the identification of large number of affected cellular processes of which as major we inferred those associated with: cytoskeleton, adhesion, cellular component organization, regulation of cell communication and intracellular protein transport. The genes were found to manifest their function mainly in cellular component such as integral components of membrane and cell periphery or different types of cell junctions. All these findings suggest that among the major cellular mechanisms affected by cryopreservation and connected with upregulation of genes are those associated with cell membrane functioning, cellular adhesion and cytoskeleton functions. It is clear from the cryobiology theory that cell membrane is under a large load during cooling and freezing processes because of osmotic stress and rapid cell shrinkage [58]. Steponkus and Wiest [59] showed that during cooling the cell shrinkage induces irreversible membrane fusion, hence the effective area of cell membrane is reduced and when returned to isotonic conditions, the tension of cell membrane is considerably increased. Moreover, cells were shown to exhibit a reversible inhibition of shrinkage at some point during a progressive increase in extracellular solute concentration, implying the development of membrane stress [60]. Another study identified such mechanisms of slow-freezing injury as macromolecule denaturation from dehydration or phase transition of membrane lipids [61]. The study of molecular events showed that slow freezing affects a membrane phase transition (liquid crystalline to gel phase), whereas rapid freezing is observed to maintain high conformational disorder of the membranes [62]. The currently applied cryoprotectants reduces these effects, however they generate osmotic stress, both during administration and post-thawing removal. So, the observed alterations in transcription levels of genes associated with cell membrane functions are probably a cellular response to all above mentioned stress factors.

It was also previously proven that cell attachment affects the biophysics of freezing. Experimental data showed that intracellular ice formation and water transport were enhanced in attached cells [62]. Liu and McGrath [63] stated that cell-cell and cell-scaffold interactions alter a cell cryopreservation response and, in turn, cellular structures involved in adhesion and intercellular contact are targets of cryopreservation-induced damage. The Authors also suggested that focal adhesions and gap junctions may support cell robustness during cryopreservation. Terry et al. [64] showed that attachment efficiency of human hepatocytes was significantly decreased after cryopreservation and gene transcripts including integrins, cadherins, catenins, and matrix metalloproteinases (MMPs) were altered by cryopreservation. All these findings suggest that cell adhesion is an important mechanism of cell viability maintenance after cryopreservation (especially for adherent MSCs) and observed changes in the expression level of genes functionally connected with adhesion and cell junctions are another important cellular response to freezing stress.

Cell adhesions can be described as a functional extension of the actin cytoskeleton [65]. The cellular cytoskeleton may have a significant role in determining post-dehydration and post-thaw cell viability, thanks to its interaction with the cell membrane. The cytoskeleton is linked almost constantly to the cell membrane [66] and is able to modify a membrane structure [67], alter its shape or morphology [68], change the mechanical properties of cells [69] and influence the membrane water transport [70]. Studies in the area of reproductive biotechnology showed that slow freezing and thawing of camel embryos resulted in a modest level of cell death but, on the other hand, more widespread disruption of the actin cytoskeleton [71]. Their findings agree with our results, which showed that the applied freezing method causes upregulation of genes associated with the regulation of the actin cytoskeleton.

The set of genes that were downregulated in post-thawing culture of the studied pig MSCs was in the vast majority associated with translation, ribosome biogenesis and respiratory processes in mitochondria with particular reference to mitochondrial envelope and its inner membrane. It is understood that changes in a gene expression level on the scale observed in this study must entail a cascade of processes responsible for gene expression regulation, purine metabolism, ribosome assembly and translation initiation. Nevertheless, the role of genes connected with mitochondria functions may be at least twofold in the cellular cryopreservation response. First, large amounts of ATP are needed for transcription and translation processes altered after cryopreservation as well as a cytoskeleton response to freezing stress. Second, it was shown that mitochondria are a target of cryopreservation induced damage [72] or may trigger apoptosis through the release of mitochondrial pro-apoptotic proteins into the cytosol [73]. In the study investigating mitochondrial respiration of DMSO cryopreserved muscle tissue which compared oxygen flux rates with respect to freshly harvested muscles, it was demonstrated that cryopreservation results in cytochrome c loss and potential disruption of the inner mitochondrial membrane [72]. A review of cellular processes occurring in cryopreserved sperm showed that the cryopreservation can induce structural damage to the mitochondria and alter biochemical processes involved in ATP production [74]. Also fragmentation damage to nuclear DNA and a diminution in sperm motility are mainly associated with damage to the structure and metabolic functioning of the mitochondrion after sperm cryopreservation. A no less important role mitochondria can play in apoptotic processes triggered after cryopreservation. It was shown that MSCs are vulnerable to cryopreservation-induced apoptosis due to the activation of apoptosis-related proteins during thawing, however, the exact relationship between cryopreservation and apoptosis is not yet well understood [75]. The complex role of mitochondria in mammalian cell apoptosis came into focus when biochemical studies identified several mitochondrial proteins that are able to activate cellular apoptotic programmes [76, 77]. In physiological conditions, these proteins reside in the intermembrane space of mitochondria and in response to a variety of apoptotic stimuli, they are released to the cytosol and/or the nucleus. They promote apoptosis either by activating caspases and nucleases or by neutralizing cytosolic inhibitors of this process [78]. The observed here coordinated cryopreservation-induced downregulation of genes connected with mitochondria functions suggests that the mitochondria play an important role in cell recovery after freezing (and their further viability) and may perform various functions depending on cryopreservation success/failure rate and cell fate decisions.

In this study we also evaluated the effect of trichostatin A administration on the transcriptome of MSCs which underwent cryopreservation. In our previous research we found that TSA is rather a gene expression stimulator in fresh MSCs and acts through the complex cellular epigenetic mechanisms [30]. The effect of TSA on frozen cells was largely similar to that observed for non-frozen cells. TSA mainly stimulated genes connected with neuron projection development, single-multicellular organism process, nervous system development, neuron development and general developmental processes and suppressed the expression of genes connected with endoplasmic reticulum functioning, intracellular protein transport, vesicle coat synthesis or G-protein coupled receptor activity. The most interesting results were, however, obtained when expression profiles of genes affected by cryopreservation and engaged in processes responsible for energy homeostasis (ATP synthesis, mitochondrial respiratory chain, cytochrome-c oxidase activity and NADH dehydrogenase activity) and mitochondrial translation were analyzed in details. TSA treatment exerted an opposite to cryopreservation stimulating effect on most of these genes, but this effect was temporary and ceased after TSA removal. This observation suggests that TSA has potential as a culture additive counteracting cryopreservation induced expression alterations, however, this statement needs further proof involving a cell phenotypical and biochemical properties analysis.

The results of this study also showed that applied cryopreservation conditions do not provoke crucial changes in the expression of known pluripotency marker genes. This is in opposition to some studies showing a loss of a variety of pluripotency markers in cryopreserved MSCs [10, 11], however, it is in line with studies showing that after cryopreservation MSCs maintain similar phenotypes, cell surface markers and growth rates in comparison to fresh cells [12, 13].

When it comes to validation of our results with qPCR, some, but in our opinion not significant, discrepancies were found which concerned two out of six analyzed genes. These discrepancies may result from yet still not accomplished annotation of the pig genome, resulting in the lack of information on all alternative mRNA splice variants. This makes design of qPCR primers representative for all transcripts difficult and may result in differences in observed overall expression levels of genes between the two applied methods.

## Conclusions

Summarizing, our results show that cryopreservation induces vast alterations in the transcriptional activity of MSCs which implies engagement in post-thawing cell recovery of processes connected with cell membrane tension or damage, cell shape maintenance, transcriptional activity and mitochondria-connected energy homeostasis or apoptosis mediation. Administration of TSA in post-thawing culture is able to a certain extent to counteract these alterations but this effect is transient and its specificity and advantages in frozen cells require further studies.

## Supporting information

S1 File Primers used for qPCR validation (T1) and results of correlation analysis between qPCR and RNA-Seq methods (T2). (DOCX)

S2 File The list of the top 1000 genes affected by cryopreservation along with their annotations. (XLSX)

S3 File The list of the top 1000 genes affected by TSA administration in the post-thawing culture along with their annotations. (XLSX)

## References

[1] Caplan AI. Why are MSCs therapeutic? New data: new insight. J Pathol. 2009; 217:318–24. doi: 10.1002/path.2469 19023885 10.1002/path.2469 PMC8793150

[2] Rho GJ, Kumar BM, Balasubramanian SS. Porcine mesenchymal stem cells–current technological status and future perspective. Front Biosci. 2009; 14:3942–61. 10.2741/3503 19273325

[3] Hoogendoorn RJ, Lu ZF, Kroeze RJ, Bank RA, Wuisman PI, Helder MN. Adipose stem cells for intervertebral disc regeneration: current status and concepts for the future. J Cell Mol Med. 2008; 12:2205–16. doi: 10.1111/j.1582-4934.2008.00291.x 18298653 10.1111/j.1582-4934.2008.00291.x PMC4514100

[4] Giannini S, Buda R, Vannini F, Cavallo M, Grigolo B. One-step bone marrow-derived cell transplantation in talar osteochondral lesions. Clin Orthop Relat Res. 2009; 467:3307–20. doi: 10.1007/s11999-009-0885-8 19449082 10.1007/s11999-009-0885-8 PMC2772930

[5] Vacanti V, Kong E, Suzuki G, Sato K, Canty JM, Lee T. Phenotypic changes of adult porcine mesenchymal stem cells induced by prolonged passaging in culture. J Cell Physiol. 2005; 205:194–201. doi: 10.1002/jcp.20376 15880640 10.1002/jcp.20376

[6] Hirose M, Kotobuki N, Machida H, Kitamura S, Ohgushi H, Tateishi T. Osteogenic potential of cryopreserved human bone marrow-derived mesenchymal cells after thawing in culture. Mater Sci Eng C. 2004; 24:355–59.

[7] Liu Y, Xu X, Ma X, Martin-Rendon E, Watt S, Cui Z. Cryopreservation of human bone marrow-derived mesenchymal stem cells with reduced dimethylsulfoxide and well-defined freezing solutions. Biotechnol Prog. 2010; 26:1635–43. doi: 10.1002/btpr.464 20572296 10.1002/btpr.464

[8] Wang X, Hua TC, Sun DW, Liu B, Yang G, Cao Y. Cryopreservation of tissue-engineered dermal replacement in Me2SO: Toxicity study and effects of concentration and cooling rates on cell viability. Cryobiology. 2007; 55:60–65. doi: 10.1016/j.cryobiol.2007.05.006 17618999 10.1016/j.cryobiol.2007.05.006

[9] Qi W, Ding D, Salvi RJ. Cytotoxic effects of dimethyl sulphoxide (DMSO) on cochlear organotypic cultures. Hear Res. 2008; 236:52–60. doi: 10.1016/j.heares.2007.12.002 18207679 10.1016/j.heares.2007.12.002 PMC2262105

[10] Reubinoff BE, Pera MF, Vajta G, Trounson AO. Effective cryopreservation of human embryonic stem cells by the open pulled straw vitrification method. Hum Reprod. 2001; 16:2187–94. 11574514 10.1093/humrep/16.10.2187

[11] Katkov II, Kim MS, Bajpai R, Altman YS, Mercola M, Loring JF, et al Cryopreservation by slow cooling with DMSO diminished production of Oct-4 pluripotency marker in human embryonic stem cells. Cryobiology. 2006; 53:194–205. doi: 10.1016/j.cryobiol.2006.05.005 16839540 10.1016/j.cryobiol.2006.05.005

[12] Goh BC, Thirumala S, Kilroy G, Devireddy RV, Gimble JM. Cryopreservation characteristics of adipose-derived stem cells: maintenance of differentiation potential and viability. J Tissue Eng Regen Med. 2007; 1:322–4. doi: 10.1002/term.35 18038424 10.1002/term.35

[13] Liu G, Zhou H, Li Y, Li G, Cui L, Liu W, et al Evaluation of the viability and osteogenic differentiation of cryopreserved human adipose-derived stem cells. Cryobiology. 2008; 57:18–24. doi: 10.1016/j.cryobiol.2008.04.002 18495102 10.1016/j.cryobiol.2008.04.002

[14] Moon JH, Kwak SS, Park G, Jung HY, Yoon BS, Park J, et al Isolation and characterization of multipotent human keloid-derived mesenchymal-like stem cells. Stem Cells Dev. 2008; 17:713–24. doi: 10.1089/scd.2007.0210 18710345 10.1089/scd.2007.0210

[15] Bhakta G, Lee KH, Magalhaes R, Wen F, Gouk SS, Hutmacher DW, et al Cryopreservation of alginate-fibrin beads involving bone marrow derived mesenchymal stromal cells by vitrification. Biomaterials. 2009; 30:336–43. doi: 10.1016/j.biomaterials.2008.09.030 18930316 10.1016/j.biomaterials.2008.09.030

[16] Liu K, Yang Y, Mansbridge J. Comparison of the stress response to cryopreservation in monolayer and three-dimensional human fibroblast cultures: stress proteins, MAP kinases, and growth factor gene expression. Tissue Eng. 2000; 6:539–54. doi: 10.1089/107632700750022189 11074940 10.1089/107632700750022189

[17] Schneider U, Mazur P. Osmotic consequences of cryoprotectant permeability and its relation to the survival of frozen-thawed embryos. Theriogenology. 1984; 21:68–79.

[18] Gao DY, Liu J, Liu C, Mcgann LE, Watson PF, Kleinhans FW, et al Prevention of osmotic injury to human spermatozoa during addition and removal of glycerol. Hum Reprod. 1995; 10:1109–22. 7657750 10.1093/oxfordjournals.humrep.a136103

[19] Mazur P. Freezing of living cells—mechanisms and implications. Am J Physiol. 1984; 247: C125–C142. doi: 10.1152/ajpcell.1984.247.3.C125 6383068 10.1152/ajpcell.1984.247.3.C125

[20] Mazur P, Cole KW. Roles of unfrozen fraction, salt concentration, and changes in cell-volume in the survival of frozen human-erythrocytes. Cryobiology. 1989; 26:1–29. 2924590 10.1016/0011-2240(89)90030-8

[21] Yang J, Diaz N, Adelsberger J, Zhou X, Stevens R, Rupert A, et al The effects of storage temperature on PBMC gene expression. BMC Immunol. 2016; 17:6 doi: 10.1186/s12865-016-0144-1 26979060 10.1186/s12865-016-0144-1 PMC4791795

[22] Riesco MF, Robles V. Cryopreservation Causes Genetic and Epigenetic Changes in Zebrafish Genital Ridges. PLoS One. 2013; 8:e67614 doi: 10.1371/journal.pone.0067614 23805321 10.1371/journal.pone.0067614 PMC3689738

[23] Oh IH, Humphries RK. Concise review: multidimensional regulation of the hematopoietic stem cell state. Stem Cells. 2012; 30:82–88. doi: 10.1002/stem.776 22083966 10.1002/stem.776

[24] Huang B, Li G, Jiang XH. Fate determination in mesenchymal stem cells: a perspective from histone-modifying enzymes. Stem Cell Res Ther. 2015; 6:35 doi: 10.1186/s13287-015-0018-0 25890062 10.1186/s13287-015-0018-0 PMC4365520

[25] Gregoretti IV, Lee YM, Goodson HV. Molecular evolution of the histone deacetylase family: functional implications of phylogenetic analysis. J Mol Biol. 2004; 338:17–31. doi: 10.1016/j.jmb.2004.02.006 15050820 10.1016/j.jmb.2004.02.006

[26] Telles E, Seto E. Modulation of cell cycle regulators by HDACs. Front Biosci. 2012; 4:831–9. 10.2741/s303 PMC3990255 22202094

[27] Choudhary C, Kumar C, Gnad F, Nielsen ML, Rehman M, Walther TC, et al Lysine acetylation targets protein complexes and co-regulates major cellular functions. Science. 2009; 325:834–40. doi: 10.1126/science.1175371 19608861 10.1126/science.1175371

[28] Yang XJ, Seto E. The Rpd3/Hda1 family of lysine deacetylases: from bacteria and yeast to mice and men. Nat Rev Mol Cell Biol. 2008; 9:206–18. doi: 10.1038/nrm2346 18292778 10.1038/nrm2346 PMC2667380

[29] Han B, Li J, Li Z, Guo L, Wang S, Liu P, et al Trichostatin A stabilizes the expression of pluripotent genes in human mesenchymal stem cells during ex vivo expansion. PLoS One. 2013; 8:e81781 doi: 10.1371/journal.pone.0081781 24312356 10.1371/journal.pone.0081781 PMC3842316

[30] Gurgul A, Opiela J, Pawlina K, Szmatoła T, Bochenek M, Bugno-Poniewierska M. The effect of histone deacetylase inhibitor trichostatin A on porcine mesenchymal stem cell transcriptome. Biochimie. 2017; 139:56–73. doi: 10.1016/j.biochi.2017.05.015 28552396 10.1016/j.biochi.2017.05.015

[31] Opiela J, Lipinski, Romanek J, Juzwa W, Bochenek M, Wilczek P. MMP-2, TIMP-2, TAZ and MEF2a transcript expression in osteogenic and adipogenic differentiation of porcine mesenchymal stem cells. Annals Anim Sci. 2016; 16:369–85.

[32] Dodt M, Roehr JT, Ahmed R, Dieterich C. FLEXBAR-Flexible barcode and adapter processing for next-generation sequencing platforms. Biology (Basel). 2012; 1:895–905. 24832523 10.3390/biology1030895 PMC4009805

[33] Kim D, Pertea G, Trapnell C, Pimentel H, Kelley R, Salzberg SL. TopHat2: accurate alignment of transcriptomes in the presence of insertions, deletions and gene fusions. Genome Biology. 2013; 14:R36 doi: 10.1186/gb-2013-14-4-r36 23618408 10.1186/gb-2013-14-4-r36 PMC4053844

[34] Anders S, Pyl PT, Huber W. HTSeq—a Python framework to work with high-throughput sequencing data. Bioinformatics. 2015; 31:166–9. doi: 10.1093/bioinformatics/btu638 25260700 10.1093/bioinformatics/btu638 PMC4287950

[35] Love MI, Huber W and Anders S. Moderated estimation of fold change and dispersion for RNA-seq data with DESeq2. Genome Biology. 2014; 15:550 doi: 10.1186/s13059-014-0550-8 25516281 10.1186/s13059-014-0550-8 PMC4302049

[36] Wang L, Wang S, Li W. RSeQC: quality control of RNA-seq experiments. Bioinformatics (Oxford, England). 2012; 28:2184–5. 10.1093/bioinformatics/bts356 22743226

[37] Mi H, Huang X, Muruganujan A, Tang H, Mills C, Kang D, et al PANTHER version 11: expanded annotation data from Gene Ontology and Reactome pathways, and data analysis tool enhancements. Nucleic Acids Res. 2017; 45(Database issue):D183–D189. 27899595 10.1093/nar/gkw1138 PMC5210595

[38] Xie C, Mao X, Huang J, Ding Y, Wu J, Dong S, et al KOBAS 2.0: a web server for annotation and identification of enriched pathways and diseases. Nucleic Acids Res. 2011; 39(Web Server issue):W316–22. doi: 10.1093/nar/gkr483 21715386 10.1093/nar/gkr483 PMC3125809

[39] Wang J, Duncan D, Shi Z, Zhang B. (2013). WEB-based GEne SeT AnaLysis Toolkit (WebGestalt): update 2013. Nucleic Acids Res. 2013; 41 (Web Server issue):W77–83. doi: 10.1093/nar/gkt439 23703215 10.1093/nar/gkt439 PMC3692109

[40] Andersen CL, Jensen JL, Ørntoft TF. Normalization of real-time quantitative reverse transcription-PCR data: A model-based variance estimation approach to identify genes suited for normalization, applied to bladder and colon cancer data sets. Cancer Res. 2004; 64: 5245–50. doi: 10.1158/0008-5472.CAN-04-0496 15289330 10.1158/0008-5472.CAN-04-0496

[41] Pfaffl MW. A new mathematical model for relative quantification in real-time RT-PCR. Nucleic Acids Res. 2001; 29:e45 11328886 10.1093/nar/29.9.e45 PMC55695

[42] Nishiyama Y, Iwanami A, Kohyama J, Itakura G, Kawabata S, Sugai K, et al Safe and efficient method for cryopreservation of human induced pluripotent stem cell-derived neural stem and progenitor cells by a programmed freezer with a magnetic field. Neurosci Res. 2016; 107:20–9. doi: 10.1016/j.neures.2015.11.011 26804710 10.1016/j.neures.2015.11.011

[43] Bourin P, Gadelorge M, Peyrafitte J-A, Fleury—Cappellesso S, Gomez M, Ragea C, et al Mesenchymal Progenitor Cells: Tissue Origin, Isolation and Culture. Transfusion Medicine and Hemotherapy. 2008; 35:160–7. doi: 10.1159/000124734 21547114 10.1159/000124734 PMC3083284

[44] Samiec M, Skrzyszowska M. High developmental capability of porcine cloned embryos following trichostatin A-dependent epigenomic transformation during in vitro maturation of oocytes pre-exposed to R-roscovitine. Anim Sci Pap Rep. 2012; 30:383–93.

[45] Samiec M, Skrzyszowska M, Opiela J. Creation of cloned pig embryos using contact-inhibited or serum-starved fibroblast cells analysed intra vitam for apoptosis occurrence. Ann Anim Sci. 2013; 13:275–93.

[46] Samiec M, Opiela J, Lipiński D, Romanek J. Trichostatin A-mediated epigenetic transformation of adult bone marrow-derived mesenchymal stem cells biases the in vitro developmental capability, quality, and pluripotency extent of porcine cloned embryos. Biomed Res Int. 2015; 2015:814686 doi: 10.1155/2015/814686 25866813 10.1155/2015/814686 PMC4381569

[47] Kątska-Książkiewicz L, Opiela J, Ryńska B. Effects of oocyte quality, semen donor and embryo co-culture system on the efficiency of blastocysts production in goats. Theriogenology. 2007; 68:736–44. doi: 10.1016/j.theriogenology.2007.06.016 17651793 10.1016/j.theriogenology.2007.06.016

[48] Kątska-Książkiewicz L, Opiela J, Ryńska B. Effects of oocyte quality and semen donor on the efficiency of in vitro embryo production in cattle. J Anim Feed Sci. 2009; 18:257–70.

[49] Marquez-Curtis LA, Janowska-Wieczorek A, McGann LE, Elliott JA. Mesenchymal stromal cells derived from various tissues: Biological, clinical and cryopreservation aspects. Cryobiology. 2015; 71:181–97. doi: 10.1016/j.cryobiol.2015.07.003 26186998 10.1016/j.cryobiol.2015.07.003

[50] Kotobuki N, Hirose M, Machida H, Katou Y, Muraki K, Takakura Y, et al Viability and osteogenic potential of cryopreserved human bone marrow-derived mesenchymal cells. Tissue Eng. 2005; 11:663–73. doi: 10.1089/ten.2005.11.663 15998208 10.1089/ten.2005.11.663

[51] Janz FDL, Debes ADA, Cavaglieri RDC, Duarte SA, Romão CM, Morón AF, et al Evaluation of distinct freezing methods and cryoprotectants for human amniotic fluid stem cells cryopreservation. J Biomed Biotechnol. 2012; 2012:649353 doi: 10.1155/2012/649353 22665987 10.1155/2012/649353 PMC3361720

[52] Ding G, Wang W, Liu Y, An Y, Zhang C, Shi S, et al Effect of cryopreservation on biological and immunological properties of stem cells from apical papilla. J Cell Physiol. 2010; 223:415–22. doi: 10.1002/jcp.22050 20082304 10.1002/jcp.22050

[53] De Rosa A, De Francesco F, Tirino V, Ferraro GA, Desiderio V, Paino F, et al A new method for cryopreserving adipose-derived and long-term cell banking technology. Tissue Eng. Part C Methods. 2009; 15:660–7. 10.1089/ten.TEC.2008.0674 19254116

[54] Tokumoto S, Sotome S, Torigoe I, Omura K, Shinomiya K. Effects of cryopreservation on bone marrow derived mesenchymal cells of a nonhuman primate. J Med Dent Sci. 2008; 55:137–43. 19845159

[55] Zhu X, Yuan F, Li H, Zheng Y, Xiao Y, Yan F. Evaluation of canine bone marrow-derived mesenchymal stem cells after long-term cryopreservation. Zool Sci. 2013; 30:1032–7 doi: 10.2108/zsj.30.1032 24320181 10.2108/zsj.30.1032

[56] Wagh V, Meganathan K, Jagtap S, Gaspar JA, Winkler J, Spitkovsky D, et al Effects of cryopreservation on the transcriptome of human embryonic stem cells after thawing and culturing. Stem Cell Rev. 2011; 7:506–17. doi: 10.1007/s12015-011-9230-1 21279480 10.1007/s12015-011-9230-1

[57] Dalman MR, Deeter A, Nimishakavi G, Duan ZH. Fold change and p-value cutoffs significantly alter microarray interpretations. BMC Bioinformatics. 2012; 13 Suppl 2:S11. 10.1186/1471-2105-13-S2-S11 PMC3305783 22536862

[58] Gao D, Critser JK. Mechanisms of cryoinjury in living cells. ILAR J. 2000; 41:187–96. 11123179 10.1093/ilar.41.4.187

[59] Steponkus PL, Wiest SC. Freeze-thaw-induced lesions in the plasma membrane In: Lyons JM, Graham D, Raison JK (eds). Low temperature stress in crop plants. Academic Press, Lond New York 1979; 231–254.

[60] Williams RJ. The mechanisms of cryoprotection in the intestinal mollusk Mylilus. Cryobiology. 1979; 4:250–62.

[61] Fishbein WN, Winkert JW. Parameters of biological freezing damage in simple solution: catalase. II. Demonstration of an optimum-recovery cooling-rate curve in a membraneless system. Cryobiology. 1978; 15:168–77. 668400 10.1016/0011-2240(78)90021-4

[62] Balasubramanian SK. Effect of cell attachment and molecular events associated with freezing biophysics and the "two factor" injury hypothesis. Retrieved from the University of Minnesota Digital Conservancy. 2011. http://hdl.handle.net/11299/119735.

[63] Liu BL. McGrath JJ. Effects of Freezing on the Cytoskeleton, Focal Adhesions and Gap-Junctionsin Murine Osteoblast Cultures. IEEE Engineering in Medicine and Biology 27th Annual Conference, Shanghai. 2005; pp.4896-4899. 10.1109/IEMBS.2005.1615570 17281340

[64] Terry C, Hughes RD, Mitry RR, Lehec SC, Dhawan A. Cryopreservation-induced nonattachment of human hepatocytes: role of adhesion molecules. Cell Transplant. 2007; 16:639–47. 17912955 10.3727/000000007783465000

[65] Dasiman R, Rahman NS, Othman S, Mustafa MF, Yusoff NJ, Jusoff WH, et al Cytoskeletal alterations in different developmental stages of in vivo cryopreserved preimplantation murine embryos. Med Sci Monit Basic Res. 2013; 19:258–66. doi: 10.12659/MSMBR.884019 24092420 10.12659/MSMBR.884019 PMC3853339

[66] Sheetz MP, Sable JE, Dobereiner HG. Continuous membrane-cytoskeleton adhesion requires continuous accommodation to lipid and cytoskeleton dynamics. Annu Rev Biophys Biomol Struct. 2006; 35:417–34. doi: 10.1146/annurev.biophys.35.040405.102017 16689643 10.1146/annurev.biophys.35.040405.102017

[67] Le Bihan T, Pelletier D, Tancrède P, Heppell B, Chauvet JP, Gicquaud CR. Effect of polar headgroup of phospholipids on their interaction with actin. J Colloid and Interface Sci. 2005; 288:88–96. 15927566 10.1016/j.jcis.2005.02.090

[68] Charras GT, Yarrow JC, Horton MA, Mahadevan L, Mitchison TJ. Non-equilibration of hydrostatic pressure in blebbing cells. Nature. 2005; 435:365–69. doi: 10.1038/nature03550 15902261 10.1038/nature03550 PMC1564437

[69] Takamatsu H, Takeya R, Naito H, Sumimoto H. On the mechanism of cell lysis by deformation. J Biomechanics. 2005; 38:117–24. 10.1016/j.jbiomech.2004.03.011 15519346

[70] Noiles EE, Thompson KA, Storey BT. Water permeability of the mouse sperm plasma membrane and its activation energy are strongly dependent on interaction of the plasma membrane with sperm cytoskeleton. Cryobiology. 1997; 35:79–92. doi: 10.1006/cryo.1997.2033 9302770 10.1006/cryo.1997.2033

[71] Skidmore JA, Schoevers E, Stout TAE. Effect of different methods of cryopreservation on the cytoskeletal integrity of dromedary camel embryos. Anim Reprod Sci. 2008; 113:196–204. doi: 10.1016/j.anireprosci.2008.07.006 18715727 10.1016/j.anireprosci.2008.07.006

[72] Pacheco S. The effect of cryopreservation on mitochondrial function in human skeletal muscle. Masters thesis, Concordia University; 2008.

[73] Gulbins E, Dreschers S, Bock J. Role of mitochondria in apoptosis. Exp Physiol. 2003; 88:85–90. 12525857 10.1113/eph8802503

[74] Figueroa E, Valdebenito I, Zepeda AB, Figueroa CA, Dumorné K, Castillo RL, et al Effects of cryopreservation on mitochondria of fish spermatozoa. Reviews in Aquaculture. 2017; 9:76–87.

[75] Bissoyi A, Pramanik K. Role of the apoptosis pathway in cryopreservation-induced cell death in mesenchymal stem cells derived from umbilical cord blood. Biopreserv Biobank. 2014; 12:246–54. doi: 10.1089/bio.2014.0005 25162461 10.1089/bio.2014.0005

[76] Verhagen AM, Ekert PG, Pakusch M, Silke J, Connolly LM, Reid GE, et al Identification of DIABLO, a mammalian protein that promotes apoptosis by binding to and antagonizing IAP proteins. Cell. 2000; 102:43–53. 10929712 10.1016/s0092-8674(00)00009-x

[77] Li LY, Luo X, Wang X. Endonuclease G (EndoG) is an apoptotic DNAse when released from mitochondria. Nature. 2001; 412:95–99. doi: 10.1038/35083620 11452314 10.1038/35083620

[78] Wang X. The expanding role of mitochondria in apoptosis. Genes Dev. 2001; 15:2922–33. 11711427

