# Supplementary material for: Evaluation of changes arising in the pig mesenchymal stromal cells transcriptome following cryopreservation and Trichostatin A treatment
Source: PLoS One. 2018 Feb 1;13(2):e0192147. doi: 10.1371/journal.pone.0192147 (PMC5794156; doi:10.1371/journal.pone.0192147)
Supplement: S1 File — (DOCX) [file pone.0192147.s001.docx]

**Table T1.** Primers used for qPCR validation.

| Gene | Strand | Sequence (5'-3') |
| --- | --- | --- |
| *LIF* | F | CCCTTTCCATCACTCCTGTC |
|  | R | CCCCTGGGCTGTGTAGTAGA |
| *FGF2* | F | TGTGCAAACCGTTATCTTGC |
|  | R | CGTTTCAGTGCCACATACCA |
| *SOX9* | F | AGAATAAGCCGCACGTCAAG |
|  | R | GCTTCTCGCTCTCATTCAGC |
| *KLF4* | F | GCCCTTAGAGGCCCACTT |
|  | R | GCAGGGCAGGATGACAGT |
| *TERT* | F | AAGACGCTGTTTGCTGTGC |
|  | R | CCGCCACGCCCTGTGGATGT |
| *NANOG* | F | TCACCAATGCCTGAGGTTTATG |
|  | R | GGGCTTGTGGAAGAATCAGG |
| *NSE* | F | TGAAGCCAAGGTGGTCATCC |
|  | R | TTGACCTCTAAGCTGTGGCG |

**Table T2.** Results of qPCR validation – expression levels detected with RNA-Seq and qPCR methods an their mutual correlations.

| **Genes** | | ***NSE*** | | ***FGF2*** | | ***TERT*** | | ***LIF*** | | ***KLF*** | | ***SOX9*** | |
| --- | --- | --- | --- | --- | --- | --- | --- | --- | --- | --- | --- | --- | --- |
| **Sample** | **Group** | **RNA-Seq** | **qPCR** | **RNA-Seq** | **qPCR** | **RNA-Seq** | **qPCR** | **RNA-Seq** | **qPCR** | **RNA-Seq** | **qPCR** | **RNA-Seq** | **qPCR** |
| 26 | Cryo | 10.6 | 1.098 | 142.6 | 0.452 | 3.2 | 0.278 | 100.0 | 1.419 | 1872.6 | 1.295 | 166.0 | 0.652 |
| 27 | TSA | 273.5 | 0.879 | 169.7 | 0.586 | 10.8 | 0.302 | 59.4 | 0.435 | 2238.5 | 1.421 | 118.9 | 0.342 |
| 28 | TSA | 483.8 | 0.380 | 220.3 | 0.378 | 13.6 | 1.093 | 21.6 | 0.059 | 3311.7 | 1.289 | 118.1 | 0.374 |
| 32 | TSA | 314.7 | 0.626 | 155.5 | 0.451 | 18.5 | 1.107 | 70.3 | 0.395 | 2608.7 | 1.493 | 79.0 | 0.216 |
| 38 | TSA>24h | 79.9 | 0.131 | 538.5 | 1.000 | 3.5 | 0.330 | 117.0 | 0.409 | 2686.5 | 0.733 | 176.0 | 0.390 |
| 47 | CTR | 1.9 | 0.827 | 22.5 | 0.023 | 2.8 | 1.685 | 120.7 | 1.354 | 2171.4 | 1.403 | 228.3 | 1.618 |
| 52 | CTR | 11.7 | 0.985 | 22.1 | 0.015 | 3.5 | 0.432 | 53.6 | 0.305 | 1422.2 | 0.590 | 275.1 | 1.181 |
| 54 | CTR | 11.1 | 0.690 | 15.1 | 0.021 | 2.0 | 1.317 | 113.7 | 1.243 | 2240.5 | 1.317 | 240.5 | 1.317 |
| 55 | CTR | 9.6 | 1.975 | 29.7 | 0.037 | 2.6 | 0.710 | 57.6 | 0.690 | 1594.8 | 1.195 | 302.2 | 1.942 |
| **Correlation coefficient** | | -0.439 | | 0.940 | | 0.106 | | 0.745 | | 0.305 | | 0.912 | |
| **Mean expression** | | 133.0 | 0.843 | 146.2 | 0.329 | 6.7 | 0.806 | 79.3 | 0.701 | 2238.5 | 1.193 | 189.3 | 0.892 |
| **Correlation fore genes (average expression)** | | 0.687 | | | | | | | | | | | |
